# Sex Work during the 2010 FIFA World Cup: Results from a Three-Wave Cross-Sectional Survey

**DOI:** 10.1371/journal.pone.0028363

**Published:** 2011-12-07

**Authors:** Wim Delva, Marlise Richter, Petra De Koker, Matthew Chersich, Marleen Temmerman

**Affiliations:** 1 South African Centre for Epidemiological Modelling and Analysis (SACEMA), Stellenbosch, South Africa; 2 International Centre for Reproductive Health (ICRH), Ghent University, Ghent, Belgium; 3 African Centre for Migration and Society, University of the Witwatersrand, Johannesburg, South Africa; 4 Centre for Health Policy, University of the Witwatersrand, Johannesburg, South Africa; Kenya Medical Research Institute - Wellcome Trust Research Programme, Kenya

## Abstract

**Background:**

In the months leading up to the 2010 FIFA World Cup in South Africa, international media postulated that at least 40,000 foreign sex workers would enter South Africa, and that an increased HIV incidence would follow. To strengthen the evidence base of future HIV prevention and sexual health programmes during international sporting events, we monitored the supply and demand of female sex work in the weeks before, during and after the 2010 FIFA World Cup.

**Methodology/Principal Findings:**

We conducted three telephonic surveys of female sex workers advertising online and in local newspapers, in the last week of May, June and July 2010. The overall response rate was 73.4% (718/978). The number of sex workers advertising online was 5.9% higher during the World Cup than before. The client turnover rate did not change significantly during (adjusted rate ratio [aRR] = 1.05; 95%CI: 0.90–1.23) or after (aRR = 1.06; 95%CI: 0.91–1.24) the World Cup. The fraction of non-South African sex workers declined during (adjusted odds ratio [aOR] = 0.50; 95%CI: 0.32–0.79) and after (aOR = 0.56; 95%CI: 0.37–0.86) the World Cup. Relatively more clients were foreign during the World Cup among sex workers advertising in the newspapers (aOR = 2.74; 95%CI: 1.37–5.48) but not among those advertising online (aOR = 1.06; 95%CI: 0.60–1.90). Self-reported condom use was high (99.0%) at baseline, and did not change during (aOR = 1.07; 95% CI: 0.16–7.30) or after (aOR = 1.13; 95% CI: 0.16–8.10) the Word Cup.

**Conclusions/Significance:**

Our findings do not provide evidence for mass-immigration of foreign sex workers advertising online and in local newspapers, nor a spike in sex work or risk of HIV transmission in this subpopulation of sex workers during the World Cup. Public health programmes focusing on sex work and HIV prevention during international sporting events should be based on evidence, not media-driven sensationalism that further heightens discrimination against sex workers and increases their vulnerability.

## Introduction

In the months leading up to the 2010 FIFA World Cup in South Africa, international media postulated that between 40,000 and 100,000 sex workers from all over the world would enter South Africa, lured by the prospects of close to half a million – many male – football fans [1], [2], [3]. Many of these fears focused on the intensification of human trafficking. A similar media hype accompanied the 2006 Soccer World Cup in Germany [4], [5]. This time, however, the speculation was augmented by fears of an increase in the incidence of HIV [6], [7], given that South Africa has amongst the highest prevalence of HIV and other sexually transmitted infections in the world [8], [9]. Consequently, numerous national and international gender, health and development agencies invested large sums of money in the provision of free condoms, HIV information campaigns for visitors and the roll-out of anti-trafficking awareness campaigns.

Yet, with the exception of a single debate in the travel medicine literature [10], [11], there has been no research published on the relationship between sex work, big sporting events, and the need for intensified HIV prevention during such events. In light of this research gap, and to strengthen the evidence base for future planning, the United Nations Population Fund (UNFPA) commissioned two studies to monitor the supply and demand of female sex work in the weeks before, during and after the 2010 Soccer World Cup. The first is a mixed-methods study focusing on street- and brothel-based female, male and transgender sex workers in three host cites, and the second is a telephonic survey among female sex workers advertising online and in local newspapers. Here, we report the findings of the latter study.

## Methods

### Design and study participants

We conducted a three-wave telephonic survey of female sex workers in the last weeks of May (pre-World Cup), June (during the World Cup) and July (post-World Cup) 2010. A sampling frame was constructed, by listing all sex worker profiles published on www.sextrader.co.za, a website with national coverage containing over 1000 profiles of sex workers. Additionally, we listed sex worker profiles published in the *adult* section of the *Classifieds* in local newspapers in the greater Johannesburg, Durban and Cape Town areas through the website www.iol.co.za. In each wave, after discarding duplicate profiles, random number tables were used to select sex workers, who were then telephonically contacted until at least 220 respondents had agreed to participate in the study. Each phone call was preceded by an SMS to the sex worker explaining the purpose of the study, and that making it clear participation was entirely anonymous and voluntary. In the telephone call, as a preamble to the invitation to participate, the research assistants explained the purpose of the study again, and emphasised its voluntary and anonymous nature. Exclusion criteria were: insufficient English language skills to understand or answer the questions, or being a male or transsexual sex worker. Eligible sex workers were asked to provide oral informed consent to survey participation. A cell phone airtime voucher of 25 ZAR (∼3.5 US$) was offered to participants, to compensate for their time spent on the interview. Responses were recorded using Epi Info 3.5.1. [12]. Participants were asked about their age; their country of origin; their current geographical work area; the number of clients in the past seven days; the country of origin of their last client; and whether a condom was used with their last client. Ethical approval for this study, including the verbal informed consent procedure, was granted by the ethics committees at Ghent University (B67020108182) and Stellenbosch University (N10/03/074).

### Statistical analysis

In the initial, descriptive data analysis, unadjusted binomial fractions, rates and means were computed, as well as surrounding exact confidence intervals, based on the Clopper-Pearson method, the chi-square distribution and the Student's t distribution respectively [13], [14], [15]. As some sex workers participated in more than one wave of the survey, we used generalized estimating equations (GEE) to test null hypotheses of no temporal changes in the weekly client turnover rate (log link function), the fractions of non-South African sex workers, and non-South African clients (logit link functions), the average age of sex workers (identity link function) and the fraction of condom-protected last sex acts with clients (logit link function) [16]. The GEE regression models took into account the effect of advertising platform (online versus newspaper) if this effect was statistically significant. All analyses were performed using the statistical package R version 2.9.0. [17].

## Results

Two weeks before the World Cup kick-off, www.sextrader.co.za listed 1098 unique profiles of female sex workers, and a total of 270 sex workers were advertising in three leading newspapers in Johannesburg, Durban and Cape Town. By the end of June, the number of unique profiles on the *sextrader* website had increased by 5.9% to 1163 and at the end of July, 1271 sex workers were advertising via this website, a further increase of 9.3%. Due to changes in the structure of the www.iol.co.za website, we were unable to monitor the number of advertisements published in the three major newspapers from Johannesburg, Durban and Cape Town.

Of 1053 sex workers contacted, 978 were eligible as 75 were excluded due to insufficient English language skills (58), or gender criteria (16 male and 1 transgender). In 260 cases, either the sex worker (239) or the receptionist/manager answering the phone (21) did not want to participate in the study, hence the overall response rate of the survey was 73.4% (718/978). Forty-seven sex workers participated in two waves of the survey while another four participated in all waves of the survey, resulting in a total sample of 663. Half of the participants (330/663) were from Johannesburg or Pretoria, while Durban (170/663) and Cape Town (163/663) each represented about a quarter of the surveyed sex workers.

**Figure 1:**
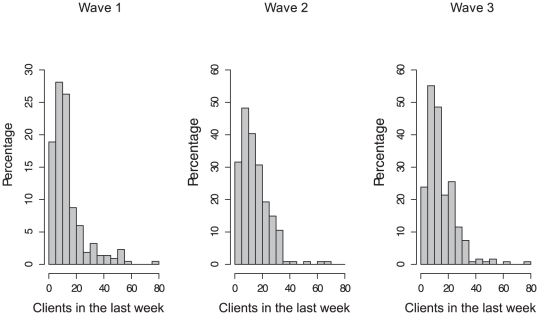
Distribution of clients in the last week before, during and after the 2010 FIFA World Cup.

At baseline, the weekly client turnover rate was 14.3 (95% CI: 13.6–15.1) for sex workers advertising in the newspapers and 11.0 (95% CI: 10.4–11.7) for sex workers advertising through the *sextrader* website. During the World Cup, these rates shifted slightly, to 14.6 (95% CI: 13.9–15.4) and 12.3 (95% CI: 11.7–13.0) respectively. Two weeks after the end of the event, the respective client turnover rates were 14.3 (95% CI: 13.6–14.9) and 12.6 (95% CI: 12.0–13.3). Figure 1 shows the distribution of clients in the last week for each of the waves of the survey.

The GEE Poisson regression model suggested no significant change in the client turnover rate during (adjusted rate ratio [aRR] = 1.05; 95% confidence interval: 0.90–1.23; P = 0.52) or after (aRR = 1.06; 95% confidence interval: 0.91–1.24; P = 0.47) the World Cup. Compared to sex workers advertising in the newspapers, those who advertised through *sextrader* had fewer clients per week (aRR = 0.83; 95% CI: 0.73–0.94; P = 0.003).

**Figure 2:**
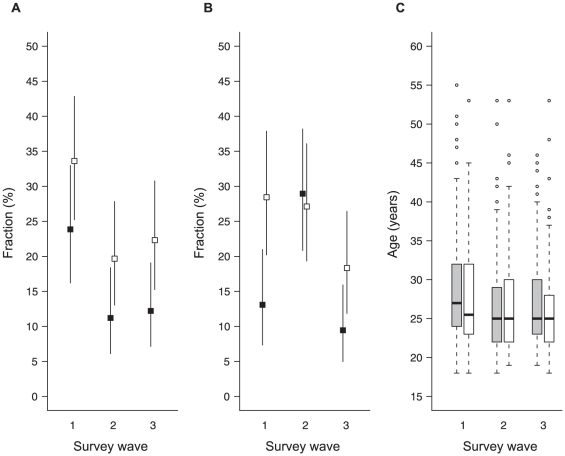
A. Fraction of non-South African sex workers, with 95% confidence intervals; B. Fraction of non-South African clients, with 95% confidence intervals; C. Box-and-whiskers plot of the age of sex workers. Black squares and gray boxes indicate sex workers advertising in the newspapers, white squares and white boxes indicate sex workers advertising on the *sextrader* website.

A relative decline of more than 40% in the fraction of non-South African sex workers was observed between the end of May and the end of June for both advertising platforms (Cf. Figure 2A). GEE logistic regression showed that the decline during and after versus before the tournament was significant (adjusted odds ratio [aOR] = 0.50; 95%CI: 0.32–0.79; P = 0.003) and aOR = 0.56; 95%CI: 0.37–0.86; P = 0.008 respectively), and that non-South African origin was associated with advertising on *sextrader* (aOR = 1.93; 95%CI: 1.31–2.84; P<0.001).

At baseline and after the World Cup, the fraction of non-South African clients was twice as high for sex workers advertising on *sextrader* compared to their counterparts who used newspaper advertising. Halfway the World Cup month, however, sex workers from both advertising platforms reported similar frequencies of non-South African origin of clients (Cf. Figure 2B). According to the regression model, the fraction of non-South African clients of sex workers advertising on *sextrader* did not change significantly during (aOR = 1.06; 95%CI: 0.60–1.90; P = 0.83) and after (aOR = 1.81; 95%CI: 0.99–3.30; P = 0.055) the World Cup, while among sex workers advertising in the newspapers, the relative increase in foreign clients during the World Cup was significant (aOR = 2.74; 95%CI: 1.37–5.48; P = 0.004).

The average age of sex workers decreased slightly from 28.6 years at baseline to 26.9 years during and after the World Cup (Cf. Figure 2C). In the unadjusted GEE model (the effect of advertising platform was not significant [P = 0.13]), this decrease was significant (P<0.01). In 2.5% of the interviews (18/718), the sex worker reported that no intercourse had taken place with her last client, and in another 14 cases, the sex worker terminated the interview before the question about condom use was asked. In six interviews, the respondent admitted not having used a condom. Four of these events were reported by respondents advertising on *sextrader*, but the unprotected sex acts were evenly spread over the three waves of the survey (2/210 at wave 1, 2/230 at wave 2 and 2/246 at wave 3), resulting in non-significant temporal changes in the GEE model for condom use during (aOR = 1.07; 95% CI: 0.16–7.30; P = 0.95) or after (aOR = 1.13; 95% CI: 0.16–8.10; P = 0.90) the Word Cup.

## Discussion

Our survey revealed a small increase in the number of sex workers advertising online during (+5.9%) and shortly after (+9.3%) the FIFA World Cup. As these changes fall well within the normal variability in the number of sex work profiles that are published on the website, our findings do not provide evidence for the massive increase in supply of sex work around the World Cup predicted by the media. Neither do the data support the widely disseminated hypothesis that thousands of foreign women and children entered South Africa – be it voluntarily or forced – to meet the increased demand in paid sex. The average age of sex workers was 1.7 years lower during the World Cup, a relatively small decrease with little or no public health or legal implications. Further, a decrease rather than an increase in the percentage of non-South African sex workers was observed in the mid-World Cup wave of the survey. During the 2006 FIFA World Cup, there was anxiety over suggestions that 40,000 women and children would be trafficked into Germany [4]. Subsequent reports found five possible cases of trafficking [18]. Similarly, South Africa feared up to 100,000 trafficking victims for 2010, but statistics recently released by the South African government included not one case of human trafficking during that World Cup [19]. The client turnover rate in our survey did not change significantly during or after the World Cup, yet the fraction of foreign clients doubled during the event among sex workers advertising in the newspaper. This may mean that a part of the local clientele of this subgroup of sex workers was temporarily replaced by foreign clients.

Besides well-known validity constraints related to self-reported outcomes, especially sexual behaviour, the main limitation of our study concerns the generalisability of the findings, given that the sampling frame only included sex workers advertising online and in newspapers. We believe that many of the respondents were brothel-based sex workers, as the telephone calls were often answered by receptionists or managers of brothels, and 48 telephone numbers were shared by a total of 115 sex workers. However, the fraction of brothels advertising online and in newspapers is unknown. Street-based sex workers are most likely underrepresented in our survey. The mixed-methods study that ran parallel to the telephonic survey, was targeted at street- and brothel-based sex workers in three World Cup host cities and complements our survey. The paper-based repeated cross-sectional survey component included face-to-face questionnaires with 1647 self-identified female sex workers. This survey found no significant changes in the demand or supply of paid sex with sex workers during the World Cup period, nor did it find significant changes in their sexual behaviour [20], [21].

As reported condom use was nearly universal throughout the study in both newspaper and online advertising sex workers, we estimate it unlikely that the slight increase in sex work during the World Cup has resulted in a considerable acceleration in transmission of HIV and other sexually transmitted infections in this subpopulation of sex workers. While the distribution of condoms and messages about safer sex might have contributed towards this success, future public health programmes focusing on sex work and HIV prevention during international sporting events such as the 2012 Olympic Games in London and the 2014 FIFA World Cup in Brazil should be based on evidence, not media-driven sensationalism that further heightens discrimination and vulnerability of sex workers.
